# Somatic, Emotional and Behavioral Symptomatology in Children during COVID-19 Pandemic: The Role of Children’s and Parents’ Alexithymia

**DOI:** 10.3390/healthcare10112171

**Published:** 2022-10-30

**Authors:** Alessia Renzi, Giulia Conte, Renata Tambelli

**Affiliations:** 1 Department of Dynamic and Clinical Psychology, and Health Studies, Sapienza University of Rome, Via degli Apuli 1, 00185 Rome, Italy; 2 Department of Human Neurosciences, Institute of Child and Adolescent Neuropsychiatry, Sapienza University of Rome, Via dei Sabelli 108, 00185 Rome, Italy

**Keywords:** children, somatic symptoms, internalizing/externalizing symptoms, alexithymia, parents, COVID-19 pandemic

## Abstract

The COVID-19 pandemic has deeply affected the psychophysical wellbeing of children worldwide. Alexithymia, a personality trait involving difficulties in identifying and expressing feelings represents a vulnerability factor for stress-related disorders. Under pandemic stress exposure, we aimed to investigate the role of parents’ and children’s alexithymia in the psychophysical symptomatology shown by children and to evaluate possible differences according to age, gender and history of COVID-19 infections. The perception of parents and children about the impact of the pandemic on children’s emotional, social and physiological wellbeing was also explored. Sixty-five familial triads were surveyed in the period from March to May 2022: children (*n* = 33 males; mean age = 9.53, sd = 1.55), mothers (mean age = 44.12; sd = 6.10) and fathers (mean age = 47.10; sd = 7.8). Both parental and children’s alexithymia scores were significantly associated with somatic and externalizing symptomatology in children. Self-reported anger and externally oriented thinking scores were higher in younger children (age 8–9.9 years) than in older ones (10–12 years). Girls scored higher than boys in somatic complaints, as reported by parents. No difference emerged between children affected/not affected by COVID-19. Notably, children reported a greater negative impact of the pandemic on their emotional and psychosocial well-being than their parents. The findings emphasize the role of alexithymia in the occurrence of psychophysical symptoms in children during the COVID-19 pandemic. The reduced parental awareness of the emotional burden imposed by the pandemic on children indicates the need to better consider how epidemics affect children’s mental health and to develop adequate preventive strategies to support them in these exceptional times.

## 1. Introduction

On 10th March 2020, with the aim to contain the spread of Coronavirus Disease 19 (COVID-19) infections, the Italian government closed all non-essential businesses and services, including schools, universities, parks, theatres and museums, and imposed severe limitations on the freedom to move and interact socially [1]. Soon thereafter, similar measures were recurrently implemented worldwide, with a crucial impact on the global economy as well as on the daily habits and quality of life of people [2]. Some categories of people may have been more vulnerable than others to the negative psychosocial effects of the pandemic and related restrictions, such as children and adolescents, who are in a critical period of their development [1,3]. For children and adolescents in Italy, the COVID-19 outbreak has represented the first great stressful community event [4] forcing them to deal with the fear of falling ill and the loss of loved ones and with the economic losses and stress of their parents, which all generated a growing feeling of uncertainty and anxiety [5].

A wealth of studies conducted over the last two years at different stages of the pandemic and in different countries have highlighted increased rates of depressive, anxiety and post-traumatic stress disorder (PTSD) symptoms and somatic complaints in children and adolescents of all ages [4,5,6,7,8,9,10,11,12]. School closures and limited outdoor leisure time have strongly restricted social interactions with peers and friends, which can represent a risk factor for children’s and adolescents’ mental health since peer relationships play a key role in their development [13]. Intuitively, limited social interaction increases feelings of loneliness, which have been repeatedly associated with an increase in mental health problems in youth during the pandemic [14,15]. More specifically, the impossibility to satisfy the need to belong to the group and to be socially connected with others can increase the risk of suicide among children and adolescents [16]. Moreover, the use of online teaching and online interactions may represent an additional source of stress for children and adolescents. Although social media have been used as a way to keep in touch with peers, this often resulted in excessive use, with increased distress, risk of victimization and social media addiction in children and adolescents [16,17].

Studies investigating the prevalent emotions experienced by children during periods of social restrictions found high rates of sadness, fear, boredom, nervousness, loneliness, sadness and anger [18,19,20,21,22,23]. Regarding parents’ perspectives, Orgiles et al. [24] found that 85.7% of parents perceived changes in their children’s emotional state and behaviors during the quarantine, and the most frequent symptoms were difficulty concentrating (76.6%), boredom (52%), irritability (39%), restlessness (38.8%), nervousness (38%), feelings of loneliness (31.3%), uneasiness (30.4%) and worries (30.1%). Furthermore, in a recent review, Amorós-Reche et al. [18] explored which socio-demographic factors may particularly have influenced the rise in emotional problems during the COVID-19 pandemic, highlighting more anxiety, depression and emotion regulation problems in females [9], in children over the age of 7, and in those coming from families with low educational and socioeconomic status [12,25]. Another study on the somatic and anxiety complains as a consequence of the pandemic [26] highlighted that children are less able to symbolize and express their emotional inner states and that their clinical manifestations of anxiety or emotional problems may more frequently include neurovegetative symptoms (e.g., tachycardia, tachypnoea, sweating and increased perspiration) and somatic symptoms (e.g., abdominal pain, hyperphagia/anorexia, nausea and headache). Therefore, in this specific population, it may be particularly relevant to investigate the somatic symptomatology considering somatization as the expression of mental distress and psychosocial stress through physical symptoms [27].

Within this context of developmental vulnerability to pandemic stress exposure, parental stress further contributes to increased emotional mood and anxiety problems in children and adolescents [18,20,24,28,29,30]. During lockdowns, children have only their parents around them to provide support, but this condition puts parents at higher risk of experiencing distress, thereby potentially impairing their ability to be supportive caregivers [31]. Emotionally focused conversations about life-threatening diseases hold important benefits for children and families’ long-term psychological well-being, but often, parents do not share their feelings or are not able to do so, possibly leading to a somatic symptomatology in their children. Therefore, the lack of parental support received by children in such difficult moments may be a reason for their more pronounced psychopathological symptoms [32].

It should be noted that the mental impact of traumatic or stressful events may be moderated by different individual characteristics such as emotion regulation capabilities [33,34,35,36]. In this direction, alexithymia, a stable personality trait involving difficulties in identifying and expressing feelings, an externally oriented style of thinking and a paucity of imagination [37,38], represents a vulnerability factor for the development of physical and mental disorders including stress-related disorders, since the lack of emotional awareness appeared to be associated with the use of ineffective coping strategies predisposing individuals to negative health consequences [34,39,40]. It has been hypothesized that elevated levels of alexithymia may be caused by exposure to dysfunctional affective environments during crucial periods for emotional and cognitive development [41]. This would hinder the child’s ability to acquire emotional awareness and to develop autonomy in recognizing and verbalizing bodily sensations and emotions [42,43,44]. Different studies have confirmed the association between alexithymia and poor physical and mental health in children and adolescents, with increased rates of both symptomatology linked to mood–emotion alterations (i.e., internalizing symptoms such as anxiety, depression, and somatic complaints) as well as to externalizing symptoms expressed in the form of behavioral dysfunctions, e.g., aggressive or rule-breaking behaviors [45,46,47,48,49,50,51]. Recently, the predictive effect of alexithymic traits on psychopathological symptoms resulting from the COVID-19 pandemic has been reported in both adult [29,52,53] and adolescent populations [54,55,56], whereas similar investigations in children are lacking.

Overall, the COVID-19 pandemic has seriously affected children’s psychological and physical wellbeing, showing high levels of emotional, behavioral and somatic symptoms. Several studies have highlighted the role played by parents in protecting or exposing children to more negative mental health consequences during the pandemic. In this light, the association between both parental and children’s alexithymia with children’s symptomatology appears to be a neglected area of investigation. Based on the above premises and literature gaps, the present study aims to explore

− The associations between children’s and parents’ alexithymia with children’s internalization and externalization of somatic symptomatology, and anger levels;
− The differences in children’s symptoms according to children subgroups based on their alexithymia mean level, age and gender and whether they contracted COVID-19;
− The comparison between parental and children’s perceptions about the impact of the pandemic on children’s emotional wellbeing and variation in social activities and physiological rhythms, with an estimation of the possible effects of COVID-19 infections within the family (child or primary caregivers) on such measures.

We hypothesized positive associations between both children’s and parents’ alexithymia with children’s symptomatology. We also expected that younger children would present a greater burden of emotional and behavioral problems because of more immature emotion regulation abilities [57] with a possible influence on resilience to stress exposure [58,59]. Moreover, we expected to find a greater symptomatology in girls than in boys, according to previous findings [18].

## 2. Materials and Methods

### 2.1. Participants, Recruitment and Procedure

The present investigation was conducted in the period between March and May 2022. The investigation was carried out in accordance with the code of ethics of the World Medical Association (Declaration of Helsinki) for experiments involving humans. Ethical approval was granted by the Ethics Committee of the Department of Dynamic and Clinical Psychology at Sapienza University of Rome.

Participants consisted of family triads (parental couple and the child) and were recruited through the snowball method in the center of Italy according to the following inclusion criteria:

− Children with an age range between 8 and 12 years old;
− Both parents available to participate;
− Parents who cohabitate with their children;
− Adequate understanding of the Italian language.

An exponential non-discriminative method was followed, i.e., after recruiting the first study participants who gave one or more referrals, each new referral then provided new contacts for referral, and so on. After obtaining individual and parent-on-child written agreement to participate, familial triads completed the study questionnaires in accordance with the research protocol at the presence of a qualified psychologist. Families in which (1) the child had a neurological disorder or pre-pandemic psychiatric diagnosis; (2) parents were separated/divorced and, therefore, not cohabitating with the child; and (3) parents had a history of psychiatric disorder were all excluded from the study. Exclusion criteria were ruled out through a clinical interview performed by an experienced clinical psychologist.

We recruited a total of 65 triads so composed: 65 children (*n* = 33 males, 51%) with average age of 9.53 (sd = 1.55; age range between minimum of 8 to maximum 12 years); 65 mothers with average age of 44.12 (sd = 6.10; age range between 32 to 55 years) and 65 fathers with average age of 47.10 (sd = 7.8; age range between 32 to 67 years). All parents were married (72.3%) or cohabitant (27.7%). Mothers most often reported their highest level of education being high school (57%) or more than high school (25%), and 55.6% were employed. As regards fathers, they most often reported their highest level of education being high school (61%) or more than high school (18.5%), and 59.6% were employed/working.

### 2.2. Measures

#### 2.2.1. Questionnaires Completed by Parents Separately

− Socio-demographic questionnaire. The questionnaire was designed to collect information concerning participants’ age, gender, educational level and parents’ occupational activity.
− 20-Item Toronto Alexithymia Scale (TAS-20) [60] is the most used self-report instrument for alexithymia evaluation in adults. It includes 20 items rated on a 5-point Likert scale, ranging from “strongly disagree” (1) to “strongly agree” (5). This instrument provides both a total score and a score for each factor. It is structured according to three factors: difficulty identifying feelings (F1) (example item: “I am often confused about what emotion I am feeling”), difficulty describing feelings (F2) (example item: “It is difficult for me to find the right words for my feelings”), and externally oriented thinking (F3) (example item: “I prefer talking to people about their daily activities rather than their feelings”). Total scores ranged from 20 to 100, with higher scores representing higher alexithymic characteristics. The questionnaire showed adequate internal reliability (total score Cronbach’s alpha = 0.75) and test–retest reliability (r = 0.83). A total Cronbach’s alpha score of 0.79 was obtained in the present study. Cronbach’s alphas of 0.86, 0.70 and 0.68 were obtained for F1, F2 and F3 scores, respectively.

#### 2.2.2. Questionnaires Completed by Parents Jointly

- Child Behavior CheckList 6–18 (CBCL 6–18) [61,62] is one of the most widely used instruments to assess child and adolescent psychopathology both in epidemiological and clinical samples. The CBCL 6–18 is a 113-item informant-report questionnaire, which asks parents to rate specific emotional–behavioral problems of their child during the past 6 months. Items are rated on a 3-point Likert scale, ranging from “0” (not true) to “2” (very true or often true), and they are grouped into eight empirically based syndrome scales: anxious/depressed, withdrawn/depressed, somatic complaints, social problems, thought problems, attention problems, rule-breaking behavior and aggressive behavior. These subscales can be combined in two broader scales: internalizing problems scale (comprising items from the anxious/depressed, withdrawn-depressed and somatic complaints scores) and externalizing problems (combining rule-breaking and aggressive behavior). Moreover, a total-problems scale comprised the scores of all the problem items. In this study, statistical analyses were performed on raw scores. Internal consistency was satisfactory for both the externalizing scale (Cronbach’s α  =  0.78) and the internalizing scale (Cronbach’s α  =  0.75).
- COVID-19 questionnaire-parent version is a questionnaire created ad hoc by the authors to explore parental perceptions regarding the impact of the pandemic on the child. The instrument contains a descriptive section to identify if the child had faced highly emotionally demanding situations during the pandemic, such as being infected by COVID-19, number of quarantines/isolation periods, number of swabs taken by the child, loss of a family member or friend due to direct effect of COVID-19 infection and number of bereavements. The questionnaire also offers a quantification of the negative impact of the pandemic on several domains of functioning of the child as rated by the parents, i.e., “Social relationships” (both intra-family relationships as well as extra-family relationships), “Physiological rhythms” (sleep and nutrition patterns) and “Emotions” (loneliness, sadness, anger and anxiety). Therefore, the section “Social Relationships” was composed of 2 items, the section “Physiological rhythms” was composed of 2 items, whereas the section “Emotions” was composed of 4 items, all of which were rated on a 3-point Likert scale (1 = not at all, 2 = a little and 3 = a lot). Greater scores highlight a greater negative impact of the pandemic on the child, as perceived by parents.

#### 2.2.3. Questionnaires Completed by the Children

- Alexithymia Questionnaire for Children (AQC) [63,64] was used to assess alexithymic features in children. The AQC is a simplified version of the original questionnaire for alexithymia for adults, the 20-item Toronto Alexithymia Scale (TAS-20; Bagby et al., 1994), and it consisted of 20 items rated on a 3-point Likert scale (0 = not true, 1 = a bit true and 2 = true) with higher score showing higher alexithymic characteristics. Similar to the TAS–20, the AQC measures the following factors: Difficulty Identifying Feelings (DIF); Difficulty Describing Feelings (DDF) and Externally Oriented Thinking (EOT). The AQC Italian version demonstrated sufficient psychometric properties (Di Trani et al., 2018), and a total Cronbach’s alpha score of 0.70 was obtained in the present study. Cronbach’s alphas of 0.70, 0.64 and 0.58 were obtained for DIF, DDF and EOT scores, respectively.
- Children’s Somatization Inventory (CSI-24) [65,66,67] was used to explore children’s perception of somatic symptoms or complaints. It included 24 items rated on a 5-point Likert scale (0 = not at all, 1 = a little, 2 = somewhat, 3 = a lot and 4 = a whole lot), reflecting the extent to which symptoms were experienced in the past 2 weeks. Higher scores indicate higher levels of somatic symptoms. This self-report measure showed adequate reliability and validity in both the original [65] and Italian version [67]. Total score Cronbach’s alpha of 0.89 was obtained in the present study.
- Children’s Inventory of Anger (ChIA) [68] is a 39-item self-report assessing the intensity of anger in children aged 6 to 16 years. Initially developed as the children’s version of the Novaco Anger Inventory (NAI) [69], the ChIA provides information on the subjective experience of anger through the evaluation of the child’s proneness to feel anger in relation to specific individual situations as well as to the source of the provocation, the person or thing involved, and the setting. It is composed of four scales: Frustration, Physical Aggression, Peer Relationship and Authority Relations. The child is asked to report on a 4-point scale “how angry (mad) you would get in that particular situation”. Vignettes with different facial expressions are used as visual aids to help the child anchor his/her ratings since impulsive/aggressive children have been found to employ pictures more often than words in their thinking. ChIA showed adequate reliability and validity in both the original version [70] as well as in the Italian version of the questionnaire [71]. A total score Cronbach’s alpha of 0.93 was obtained in the present study. Cronbach’s alphas of 0.79, 0.86, 0.75 and 0.81 were obtained for Frustration, Physical Aggression, Peer Relationship and Authority Relations, respectively.
- COVID-19 questionnaire-child version is a questionnaire created ad hoc by the authors to explore the child’s perception regarding the impact of the pandemic and related social restrictions on different dimensions of his/her life. Specifically, the questionnaire was designed to cover the same domains of functioning explored by the parent-version of the COVID-19 questionnaire (i.e., variations in the child’s social relationships, physiological rhythms and emotions). Children were asked to report if the COVID-19 pandemic had had a negative impact on their relationships with peers and parents; on their sleep; on their food intake; and on their levels of sadness, anxiety, anger and loneliness by answering questions on a 3-point Likert scale (1 = not at all, 2 = a little and 3 = a lot). Greater scores highlight a greater negative impact of the pandemic perceived by the child.

### 2.3. Statistical Analyses

All statistical analyses were executed using the Statistical Package for Social Science version 25 for Windows (SPSS version 25; IBM, Armonk, NY, USA). Data were reported as frequency and percentage for discrete variables and as means and standard deviations for continuous variables. Pearson’s correlation analysis was computed to measure associations between children’ and parents’ variables and dimensions investigated. *T*-tests for paired sample were performed to evaluate possible differences between parents’ and child’s evaluation of the impact of COVID-19 on the child’s relational, physiological and emotional well-being. One-way ANOVAs were performed to explore possible differences in the investigated psychological dimensions between groups of children created according to following variables: gender (females/males), age (8–9.9 years/10–12 years), ACQ scores (below/above mean value of the sample) and having contracted a COVID-19 infection (yes/no). Multivariate factorial analyses were also conducted to evaluate the effect of COVID-19 infections within the family environment of the child on his/her emotional well-being, relational well-being and physiological rhythms variations by considering the following as independent variables: (i) former history of COVID-19 infection in the child, (ii) COVID-19 infections in primary caregivers and (iii) the experience of quarantine periods by the child. The alpha level for statistical significance was set at *p* < 0.05.

## 3. Results

Table 1 shows children’s characteristics on the psychological dimensions investigated, whereas in Table 2, parental alexithymic characteristics have been reported.

In total, 53.8 % of children (*n* = 35) had not been affected by COVID-19, while 46.2% (*n* = 30) had. Parents reported bereavements only in 7.7% of cases, while 92.7% of the sample did not report losses caused by the virus. All children had been through several quarantine periods and a mean number of swab tests of 9.25 (sd = 5.25; range between 3 to 20). 

Children’s ChIA scores were organized according to cut-off criteria, only 32.3% of children (*n* = 21) reported a score in the normal range (score range between 40 to 59) whereas 67.8% of children (*n* = 44) reported a score in the clinical range, specifically a score over (score range between 60 to 69) and greatly over (scores ≥ 70) the normal range, highlighting high levels of perceived anger. 

Children’s alexithymia mean scores (36.86; sd = 4.86) appeared to be in line with those reported in the Italian general population of children aged from 8 to 14 years (37.65; sd = 5.70) [42]. Additionally, both mothers’ and fathers’ alexithymia mean scores (Table 2) appeared to be in line with those reported by Italian general population (m = 44.7; sd = 11.3) [60].

As regards gender differences on psychological symptoms, the only significance obtained was on CBCL 6–18 Somatic Complaints in the direction of higher scores in girls than boys [F = 5.938; *p* = 0.02, η^2^ = 0.09]. Differences between the subgroups of children with ACQ scoring below/above the sample’s mean ACQ value (m = 36.86, sd = 4.86) were evaluated. Twenty-seven children had scores below the mean value (m = 32.38, sd = 2.65), and thirty-eight children had scores above the mean value (mean = 39.83, sd = 2.21). A significant difference emerged on CSI scores in the direction of higher scores in the group of children with higher alexithymia scores (F = 5.395; *p* = 0.02, η^2^ = 0.08). Broader differences according to age groups emerged. More specifically, children in the age range 8–9.9 years scored significantly higher on the ChIA Total [F = 4.103; *p* = 0.047, η^2^ = 0.06], Frustration [F = 7.328; *p* = 0.009, η^2^ = 0.12] and Physical Aggression [F = 4.616; *p* = 0.036, η^2^ = 0.07] scales and on ACQ external oriented thinking [F = 7.193; *p* = 0.009, η^2^ = 0.10] than children in the age range 10–12 years (see Table 3). No difference emerged between groups of children affected/not affected by the virus on the different psychometric scales. 

Children’s ACQ total scores were positively associated with CSI total score (r = 0.314; *p* = 0.012), CBCL 6–18 Externalizing Problems score (r = 0.292; *p* = 0.020) and CBCL 6–18 Aggressive Behavior score (r = 0.304; *p* = 0.015) scores. The ACQ subscale “difficulties in identifying feelings” was positively associated with CSI total score (r = 0.425; *p* = 0.001) and CBCL 6–18 Aggressive Behavior score (r = 0.250; *p* = 0.04). No associations between the other two subscales of the ACQ (“difficulties in describing feelings” and “external orientated thinking”) emerged with respect to psychological symptoms. 

As regards the relation between mothers’ alexithymia scores with children’s symptomatology, few associations were detected. More specifically, TAS-20 “difficulties in identifying feelings” was positively associated with CBCL 6–18 Somatic Complaints (r = 0.259; *p* = 0.04) and Internalizing Problems (r = 0.280; *p* = 0.026). TAS-20 “externally orientated thinking” was instead negatively associated with ChIA physical aggression (r = −0.245; *p* = 0.04), whereas TAS-20 total score was positively associated with CBCL 6–18 Internalizing Problems (r = 0.261; *p* = 0.039). 

Associations between fathers’ alexithymia scores and children’ symptomatology substantially overlapped with those of mothers. Indeed, TAS-20 “difficulties in identifying feelings” was positively associated with CBCL 6–18 Somatic Complaints (r = 0.404; *p* = 0.001) and Internalizing Problems (r = 0.263; *p* = 0.04), TAS-20 “difficulty in describing feelings” was positively associated with Somatic Complaints (r = 0.377; *p* = 0.003), whereas TAS-20 total was positively associated with CBCL 6–18 Internalizing Problems (r = 0.340; *p* = 0.007) and Somatic Complaints (r = 0.440; *p* = 0.001). The correlation analysis between child alexithymia and parental alexithymia failed to show any significant association of the child’s score with either the mother’s or father’s scores.

As regards the impact of the pandemic on children’s emotional, relational and physiological domains, there was a significant difference between the evaluation done by parents and children (see Table 4). 

Specifically, children reported greater negative impact on their emotional well-being [t = −2.33; *p* = 0.023; r = 0.28] and physiological rhythms [t = −7.028; *p* = 0.001; r = 0.66] compared to those reported by their parents, whereas no difference emerged between parental and children ratings about the impact on the child’s relational patterns. Moreover, no effect of individual or familial COVID-19 infection history or of quarantine periods was detected on the ratings of the pandemic’s impact, as revealed by multifactorial analysis of variance.

## 4. Discussion

In the context of over two years into the outbreak of COVID-19, the mechanisms for the association between pandemic exposure and mental health outcomes in the general population, particularly in children, remain largely unknown. In children, both individual characteristics (such as emotional competencies) and parental emotional capabilities have been highlighted as potential moderators of psychopathological outcomes resulting from stressful events [33,34,35,36]. 

As regards the relationship between children’s alexithymia levels and psychopathological symptoms during the pandemic, lower emotional capabilities (higher alexithymia) were associated with higher somatization problems, both self-reported (CSI-24) as well as described by the parents (CBCL 6–18 Somatic Complaints). This is highly consistent with a variety of studies establishing a clear link among somatization, physical illness and alexithymia, both in adults [72,73,74,75] as well as in children and adolescents [67,76,77,78,79]. The results from the current study further support the model proposed by Rieffe et al. [80], showing that children who have problems in differentiating emotions may more likely rely on physical solutions in stressful events because of difficulties in coping with their emotional responses to an overwhelming stressor. Moreover, children’s alexithymia positively correlated with CBCL 6–18 externalizing problems (r = 0.292; *p* = 0.020) and aggressive behaviors (r = 0.304; *p* = 0.015). The inability to identify one’s own emotions may facilitate aggressive behavior following a triggering emotional situation [81,82] and numerous studies found that alexithymia is positively associated with verbal and physical aggression [83,84,85], with a mediation effect played by impulsivity [86]. 

A further objective of the present study was to analyze the correlation between parents’ alexithymia with children’s symptomatology during the pandemic. In line with our hypothesis, we found significant correlations between both mothers’ (r = 0.259; *p* = 0.04) and fathers’ (r = 0.404; *p* = 0.001) TAS-20 “difficulties in identifying feelings” subscale with the CBCL 6–18 Somatic Complaints subscale, pointing out that parents’ difficulties in identifying their own feelings may be related to parents’ perception of somatic problems severity in their child. Children in families characterized by low emotional expressiveness and alexithymia more frequently undergo hospitalizations [87] and present debilitating pain syndromes [88]. Several mechanisms could explain this finding. First, alexithymia has been inversely associated with reflective functioning [89,90,91], namely the ability to understand one’s own and other’s behaviors as the result of underlying mental states. Parents with alexithymia may, therefore, lack the ability to interpret and promote the expression of the emotional states of their child, which would be crucial, in turn, to allow him/her to develop the same capacity [92] and rely less on somatization to downregulate negative affects. Second, children’s symptoms are also influenced by parental reinforcement [93,94], and alexithymic parents may indirectly strengthen children’s somatic complaints by predominantly allocating attention to bodily issues while neglecting the child’s emotional functioning and needs. Third, recent research has emphasized the role of parent–child discussion on the pandemic as an important protective factor against psychopathological outcomes, showing that children and adolescents who discussed the pandemic with their parents were less likely to report symptoms of depression, anxiety and stress [95]. Although not directly tested in our study, an intriguing possibility is that parental alexithymia has contributed to a lack within families of emotionally focused conversations about COVID-19 and related life changes, thus promoting somatic symptoms in children as a way to express distress when emotional attunement and support from parents are unavailable. The outlined hypotheses await future investigations to better elucidate which mechanisms provide the best explanation for the association between parental alexithymia and children’s somatic symptoms.

Parental alexithymia was also significantly associated with greater parental perception of internalizing and depressive problems in their child (mothers: r = 0.261; *p* = 0.039; fathers: r = 0.340; *p* = 0.007). Similarly, Davodi-Boroujerd et al. [96] showed that maternal alexithymia can act as relevant factor in the development of internalizing problems in children. Contingent stress imposed on parents by the pandemic may further have played a role in this regard, as highlighted by an increased maternal perception of internalizing symptoms in children after the COVID-19 outbreak compared to the pre-COVID-19 era [97]. Moreover, maternal stress due to COVID-19-related restrictions has been associated with increased depressive symptoms and decreased positive parenting behaviors with negative influence on children’s internalizing and externalizing problems [98]. 

As specifically regards a different symptomatologic expression according to age, younger children (age range 8–9.9 years) exhibited higher levels of alexithymia, perceived anger and problems in regulating aggressive behavior as compared to children aged 10–12 years. It is presumable that younger children are less able to symbolize and describe their subconscious emotional states, as further supported by the developmental aspects of alexithymia, which decreases according to age [99]. Age may also play an important role in anger experience and expression, as it is well-known that across age groups, anger regulation is influenced by the child’s cognitive and language capabilities, and social environment [100]. Younger children tend to engage more often in confrontational anger behaviors, whereas adolescents tend to express their anger less outwardly [101,102]. With increasing age, children may also repress their anger more often because anger is seen as less socially acceptable [103]. Thus, our results point out that younger children may represent a particularly vulnerable group under pandemic exposure because of greater difficulties in recognizing and regulating negative emotional states. 

Lastly, the present study was designed to explore levels of accordance between children’s and parents’ perceptions about the impact of the COVID-19 pandemic on the emotional and relational well-being of children. Parents and children shared similar views on the limitations imposed by the pandemic on the child’s relational/social life (both intra-family and extra-family relationships), but they significantly diverged in the evaluation of its impact on emotional well-being (loneliness, sadness, anger and anxiety) and physiological rhythms (sleep and nutrition patterns), which were both rated as more severely affected by children than by parents. Adults’ underestimation of the burden imposed by the pandemic on child’s well-being may derive from their own pandemic-related economic and health concerns, which may have affected their ability to intercept signals of distress in their child. Another possible explanation is that latent parental emotional difficulties may have worsened the capability to recognize the pandemic burden on children’s emotional and physiological dimensions. Interestingly, children with and without a history of COVID-19 infection were almost equally represented in our sample (46.2% vs. 53.8%, respectively) and factorial analysis showed no influence of former infections in the child or primary caregivers on the variation of psycho-social wellbeing of children during the pandemic. These findings support the possibility that the pandemic per se as cumulative trauma load [104] rather than the specific experience of COVID-19 infections may have greatly influenced the level of emotional and behavioral symptoms reported by children. 

Several limitations should be considered in interpreting these results. First, sample enrollment methodology and restricted size may limit the generalizability of the results to the national and general population. Studies with a broader sample size should be realized. Second, the use of a self-report measure may introduce biases related to social desirability and/or text comprehension, the latter especially for children. In this direction future investigation should include a clinician report instrument for overcoming these limits. Third, additional psychosocial factors relevant for children’s mental health (e.g., peer relationship, academic performance, academic pressure and poor family functioning) [56] were not included in this study. Thus, the results of this study could not be controlled for the possibility of residual confounding caused by unmeasured variables. Therefore, additional psychological factors relevant for children’s psychological wellbeing should be considered in future studies to reduce potential confounding effects. Lastly, the cross-sectional design of the study hinders the possibility to draw causal conclusions on the observed relationships. Longitudinal studies appeared to be necessary and several follow-up studies could be important to observe the associations found over time.

## 5. Conclusions

Within the context of these limitations, our findings emphasize the role of alexithymia in the occurrence of somatization and externalizing problems in children during the pandemic. Our study also raises the possibility that parental alexithymia may further contribute to the level of somatic complaints and internalizing problems experienced by children in this difficult context. Therefore, alexithymia should be considered as a vulnerability factor in the etiology of trauma-related mental health problems in children, and younger children may specifically represent a particularly vulnerable group because of greater difficulties in recognizing and regulating negative emotional states. Notably, our study highlights a reduced parental awareness of the emotional burden imposed by the pandemic on children, which demands future attention from health authorities, researchers, professionals and the general community. Interventions focused on parent–child relationships may be crucial in reducing the negative impact of the current health crisis on children’s wellbeing and improving children’s adaptation strategies to possible future stressful life events. Promoting more sensitive parenting can improve the sense of security in children and the adoption of useful coping strategies to face difficult or traumatic events [53]. It is essential for health policies to better consider the special needs of all children in these exceptional times and to develop adequate preventive strategies to actively promote their well-being. In this direction the launching of prevention campaigns on the impact of isolation and loneliness on children’s mental and physical health and the related risk to develop social media addiction are recommended. Furthermore, considering of the accumulating evidence regarding social limitation-imposed negative impact on children’s mental health and the lower rates of long-term negative consequences of COVID-19 infections in this age group, a more cautious approach in the application of social restrictions to this specific population should be considered to safeguard children’s mental health in the case of future health crises.

## Figures and Tables

**Table 1:** Children’s psychological characteristics.

	M	SD
AQC Total	36.86	4.85
AQC Difficulty in Identifying Feelings	11.85	3.04
AQC Difficulty in Describing Feelings	9.38	2.07
AQC External Oriented Thinking	15.63	2.25
CSI Total	20.07	14.35
ChIA Total	102.46	20.19
ChIA Frustration	25.49	6.14
ChIA Physical Aggression	27.23	5.92
ChIA Peer Relationships	22.37	5.29
ChIA Authority Relations	27.93	6.22
CBCL 6–18 Anxious/Depressed	4.52	3.42
CBCL 6–18 Withdrawn/Depressed	1.56	1.40
CBCL 6–18 Somatic Complaints	1.33	1.51
CBCL 6–18 Social Problems	2.70	1.80
CBCL 6–18 Thought Problems	1.93	1.90
CBCL 6–18 Attention Problems	3.65	2.70
CBCL 6–18 Rule-breaking Behavior	1.38	1.40
CBCL 6–18 Aggressive Behavior	4.57	4.01
CBCL 6–18 Internalizing Problems	7.38	4.83
CBCL 6–18 Externalizing Problems	5.95	4.80

Note: AQC = Alexithymia Questionnaire for Children; CSI = Children’s Somatization Inventory; ChIA = Children’s Inventory of Anger; CBCL6–18 = Child Behavior CheckList 6–18. Data are presented as mean (M) and standard deviation (SD).

**Table 2:** Parental alexithymic characteristics.

	M	SD
Mothers		
TAS-20 Total	41.53	9.48
TAS-20 Difficulty in Identifying Feelings	13.35	5.03
TAS-20 Difficulty in Describing Feelings	11.70	3.83
TAS-20 External Oriented Thinking	16.49	4.08
Fathers		
TAS-20 Total	44.25	10.30
TAS-20 Difficulty in Identifying Feelings	13.36	5.82
TAS-20 Difficulty in Describing Feelings	12.89	4.15
TAS-20 External Oriented Thinking	18.00	4.30

Note: TAS-20 = 20-item Toronto Alexithymia Scale.

**Table 3:** Significant differences in psychological characteristics in children aged 8–9.9 vs. children aged 10–12 years.

	Children 8–9.9 Years Old		Children 10–12 Years Old		F	*p*	η^2^
	M	SD	M	SD			
ChIA Total	108.03	24.27	98.15	13.58	4.103	0.047	0.06
ChIA Frustration	27.64	7.10	23.69	4.30	7.328	0.009	0.12
ChIA Physical Aggression	28.97	6.94	25.95	4.05	4.616	0.04	0.07
AQC External Oriented Thinking	16.41	1.72	15.00	2.42	7.193	0.009	0.10

Note: AQC = Alexithymia Questionnaire for Children; ChIA = Children’s Inventory of Anger.

**Table 4:** Differences between children’s and parents’ evaluation of the impact of the pandemic on children.

	Children’s Evaluation		Parents’ Evaluation		t	*p*	Effect Size
	M	SD	M	SD			
Social relationships	4.22	0.78	4.42	1.31	1.045	0.300	-
Physiological rhythms	4.17	0.78	3.00	1.10	−7.028	0.001	0.66
Emotions	9.20	2.12	8.45	2.18	−2.334	0.023	0.28

## Data Availability

The data that support the findings of this study are available from the corresponding author upon request.
